# Time-resolved molecular measurements reveal changes in astronauts during spaceflight

**DOI:** 10.3389/fphys.2023.1219221

**Published:** 2023-07-14

**Authors:** Minzhang Zheng, Jacqueline Charvat, Sara R. Zwart, Satish K. Mehta, Brian E. Crucian, Scott M. Smith, Jin He, Carlo Piermarocchi, George I. Mias

**Affiliations:** ^1^ Department of Biochemistry and Molecular Biology, Michigan State University, East Lansing, MI, United States; ^2^ Institute for Quantitative Health Science and Engineering, Michigan State University, East Lansing, MI, United States; ^3^ KBR, Houston, TX, United States; ^4^ University of Texas Medical Branch, Galveston, TX, United States; ^5^ NASA Johnson Space Center, Houston, TX, United States; ^6^ Department of Physics and Astronomy, Michigan State University, East Lansing, MI, United States

**Keywords:** astronaut, microgravity, time series, immune response, metabolites, nutrition

## Abstract

From the early days of spaceflight to current missions, astronauts continue to be exposed to multiple hazards that affect human health, including low gravity, high radiation, isolation during long-duration missions, a closed environment and distance from Earth. Their effects can lead to adverse physiological changes and necessitate countermeasure development and/or longitudinal monitoring. A time-resolved analysis of biological signals can detect and better characterize potential adverse events during spaceflight, ideally preventing them and maintaining astronauts’ wellness. Here we provide a time-resolved assessment of the impact of spaceflight on multiple astronauts (n = 27) by studying multiple biochemical and immune measurements before, during, and after long-duration orbital spaceflight. We reveal space-associated changes of astronauts’ physiology on both the individual level and across astronauts, including associations with bone resorption and kidney function, as well as immune-system dysregulation.

## 1 Introduction

As human space exploration continues to expand, we anticipate an increasing number of long-duration and deep space missions. Beyond missions in low Earth orbit (LEO), plans are currently underway for a return to the moon, and utilizing such missions towards building a gateway for inter-planetary travel to Mars. Half a century of long-duration spaceflights on space stations has already demonstrated that such missions will pose several health risks to astronauts that have to be addressed. There are five categories of hazards faced by astronauts, that can directly contribute to health risks (Afshinnekoo et al., 2020; Patel et al., 2020): (i) Transitioning from Earth’s gravity to effective weightlessness or lower-than-Earth gravity. (ii) Experiencing higher levels of radiation, with considerably less shielding in the absence of Earth’s protective magnetic field. (iii) Being in isolation during long-duration missions. (iv) Living in a closed environment with an evolving ecosystem for long periods of time. (v) Traveling and living at vast distances from Earth, where communications and facilities will be limited for direct healthcare assessment and intervention if necessary. The multiple physiological effects associated with these hazards include muscle and bone weakening, kidney stone risks and other problems due to fluid redistribution in the upperbody, cancer risks due to radiation, immune dysregulation, cognitive and behavioral effects, and nutritional deficits (Crucian et al., 2016; Baker et al., 2019; Patel et al., 2020). All these hazards and associated risks dynamically change during space missions and necessitate close monitoring of crew members’ health to ensure their safety and wellbeing.

Astronauts have experienced multiple clinical symptoms in long-duration LEO flights, with notable medical events reported in up to 46% of the crew members (Crucian et al., 2016). The most frequently reported events (defined as involving symptoms that are recurring or have prolonged duration, or are unresponsive to treatment), included cold sores, rashes, allergies, and infectious diseases (Crucian et al., 2016). Several studies aim to understand the molecular basis of these medical symptoms in long flights. Krieger et al. (Krieger et al., 2021) investigated cytokines in plasma and saliva in 13 astronauts during 4–6 months International Space Station (ISS) flights, and identified persistent immune dysregulation. Nutritional deficits have also been directly linked to space-related clinical symptoms and systemic physiological changes (Smith et al., 2021). Smith et al. evaluated astronaut nutritional status during long stays on the ISS, identifying multiple marker changes associating bone resorption and oxidative damage across 11 astronauts (Smith S. M. et al., 2005). Given the mission-critical role of maintaining astronaut health, the National Aeronautics and Space Administration (NASA) has developed the integrated medical model (IMM) as a tool for identifying medical conditions and quantifying medical risks to astronauts (Keenan et al., 2015; Crucian et al., 2016; Antonsen et al., 2022). Furthermore, the monitoring of standard medical physiological measurements, immune and metabolite markers, is now being supplemented with an expanding array of thousands of molecular measurements (omics) in individual astronauts. This was exemplified by the astronaut Twins Study (Garrett-Bakelman et al., 2019), which focused on molecular biology measurements to compare the effects of spaceflight on two twins, one on a 340-day mission, while the other was observed during this period on Earth. Multiple changes were observed giving a glimpse of how long-duration spaceflight affects an individual astronaut’s physiology.

**FIGURE 1:**
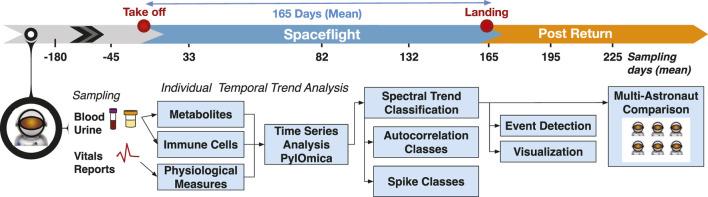
Study overview. The study followed 27 astronauts across multiple long-duration missions (adjusted mean 165 days) on ISS. The data included multiple time measurements from 180 days pre-flight to 2 months post-flight for different blood and urine-detected molecules, as well immune cell characterizations, physiological measurements, and medical reports. The time-resolved measurements were analyzed per astronaut to identify temporal trends and detect events of interest. The individual measurements were finally used to construct a multi-astronaut comparison based on similar temporal changes across measurements.

The next steps must enable monitoring of data from multiple astronauts during long-duration space missions, and should include the implementation of a personalized approach that can establish baseline characteristics in each crew member, detect deviations therefrom, and detect potentially adverse medical events. Such personalized monitoring of health based on omics has been implemented in the NASA Twins Study (Garrett-Bakelman et al., 2019), and on Earth, using blood (Chen et al., 2012), urine, saliva (Mias et al., 2021) and fecal samples. Testing methods have included microbiomes (Zhou et al., 2019), personalized coaching (Price et al., 2017) and digital devices (Li et al., 2017). In this investigation, we implemented the first (to our knowledge) multi-astronaut individual-focused monitoring in long-duration spaceflight. We characterized personal biological signals in 27 astronauts (8 female, 19 male) and assessed multiple metabolites, immune cell constitution, and physiological measurements spanning long-duration orbital missions (mean flight duration ∼165 days), Figure 1. Using spectral-based methods (Mias et al., 2016; Domanskyi et al., 2019; Mias et al., 2021) we identified temporal trends, and classified multi-signal responses corresponding to in-flight and return to Earth changes. Our analysis addressed typical spaceflight research limitations, including monitoring issues that include uneven time sampling and missing data, as well as different sampling rates for different astronauts. We identified a set of time-resolved changes in metabolites and immune cell behavior that was concordant across the majority of astronauts, and associated with bone resorption, renal stone formation, T cell and monocyte dysregulation and nutritional deficiencies. Finally, we constructed a network assessing inter-astronaut similarities, identifying groups of astronauts with similar time-resolved trends and reported clinical symptoms. Our findings identify significant metabolite and immunological modifications during spaceflight, and suggest that individual-focused monitoring must be implemented to identify the onset of these changes in order to also address them in a timely manner with personalized countermeasures.

## 2 Methods

### 2.1 Ethical approval

All protocols described herein were reviewed and approved by the NASA Institutional Review Board (NASA eIRB study ID: STUDY00000201) and by Michigan State University (MSU) Biomedical and Health Institutional Review Board (MSU study ID: STUDY00003581) in accordance with the requirements of the Code of Federal Regulations on the Protection of Human Subjects. Written informed consent was obtained from all participating subjects (astronauts). All methods described in this investigation were carried out in accordance with the relevant guidelines and regulations. Additionally, approvals for data use and manuscript/data submission for the study were obtained from the Lifetime Surveillance of Astronaut Health (LSAH) advisory board.

### 2.2 Subjects

Forty-seven potential subjects were identified as United States (US) astronauts that flew across 32 ISS and space shuttle expeditions and participated in two studies on Nutritional Status Assessment (SMO 016E), and Validation of Procedures for Monitoring Crewmember Immune Function (SMO 015). Thirty-eight astronauts agreed to participate following informed consent, and the analysis presented in this manuscript focuses on 27 subjects (19 male and 8 female) for whom long-duration measurements on the ISS were available (mean-based duration 165 days). Data for these subjects were obtained from LSAH and the Life Sciences Data Archive (LSDA).

### 2.3 Data and preprocessing

The data from the investigation include the characteristics that were assessed in SMO 015 and SMO 016E. The characteristics/measurements are listed at the respective study records on the NASA Life Sciences Portal (NLSP), which include additional information for each of the studies (the links provided in the references for SMO 015 (Sams et al., 2005) and SMO 016E (Smith S. et al., 2005), including experiments, images and relevant literature. The metabolic and immune assays used have been summarized by Douglas et al. (2022).

The raw data included all the timepoints measured for each astronaut, and as astronaut stay duration times may be unique to specific astronauts, such data may be attributable. To create non-attributable (deidentified) data, for each astronaut the different measurements were summarized into a maximum of 8 timepoints (using averages of measurements within each time bin). These measurement time bins were labeled as −180 and −45 days for pre-flight; early flight (33 days), mid-flight (82 days) and late flight (132 days) measurements; return (165 days), return+30 days (195 days) and return + 60 days (225 days). The binned data were then used for two comparisons: (I) individual astronaut analysis and (II) and across-astronaut comparison.

### 2.4 Individual astronaut analysis

#### 2.4.1 Time series categorization

The data from each individual astronaut were analyzed using PyIOmica in Python, and the calculateTimeSeriesCategorization function (Domanskyi et al., 2019). Here for each astronaut *a*, and each type of measurement *m*, the time series *X*_*ma*_ was analyzed, at timepoints *t*_*i*_ ∈ { − 180, − 45, 33, 82, 132, 165, 195, 225} days. For each *X*_*ma*_ the difference in intensity of each timepoint *i* was computed relative to the pre-flight timepoint (−45 days) intensity, ${\tilde{X}}_{ma}\left(t_{i}\right)=X_{ma}\left(t_{i}\right)-X_{ma}\left(-45\right)$ . Finally, $\tilde{X_{m}a}$ was normalized using Euclidean norm to *Q*_*ma*_, where *Q*_*ma*_ (*t*_*i*_) = $\frac{{\tilde{X}}_{ma}\left(t_{i}\right)}{\sqrt{\sum_{j}\left({\tilde{X}}_{ma}{\left(t_{j}\right)}^{2}\right)}}$ .

The calculateTimeSeriesCategorization algorithm uses spectral methods to classify time series (Domanskyi et al., 2019; Mias et al., 2021): Internally, the Lomb-Scargle periodogram *P*_*ma*_(*f*) of each time series is computed at a series of frequencies *f*. The inverse Fourier transform of *P*_*ma*_(*f*) yields the autocorrelations, {*ρ*_*mal*_}, at lags *l* ∈ {0, … , *N*/2}, where N is the number of timepoints. A bootstrap time series set *B*_*ja*_(*t*), with *j* ∈ {1, … , 10^5^} is also generated (by sampling with replacement across the time series of each astronaut). By computing the autocorrelations of each *B*_*ja*_(*t*), null distributions at each lag *l*, and the corresponding 0.95 quantiles {*ρ*_*ql*_} are obtained. These quantiles are used to assign classes [*Lag M*], where *M* is the smallest lag for which the autocorrelation at lag *l* of a signal *X*_*ma*_ is greater than the corresponding bootstrap distribution quantile:

$$M=\text{Min}\left[\left\{l:\rho_{\text{mal}}\geq \rho_{\text{ql}}\right\}\right].$$ (1)

The algorithm also checks whether time series *X*_*ma*_ that do not fulfill the criteria in Eq. 1 have any pronounced intensity maxima or minima. Using again a bootstrap set of time series, the 0.95 quantiles max_*qN*_ and min_*qN*_ are computed, from the distributions of the maxima and minima of the bootstrap time series respectively. If max (*X*_*ma*_) > max_*qN*_, *X*_*ma*_ is assigned to class [*SpikeMax*]. If min (*X*_*ma*_) < min_*qN*_, *X*_*ma*_ is assigned to class [*SpikeMin*]. Time series assigned to any of the classes [*LagM*], [*SpikeMax*], and [*SpikeMin*] are considered to display statistically significant trends.

#### 2.4.2 Cluster categorization

**FIGURE 2:**
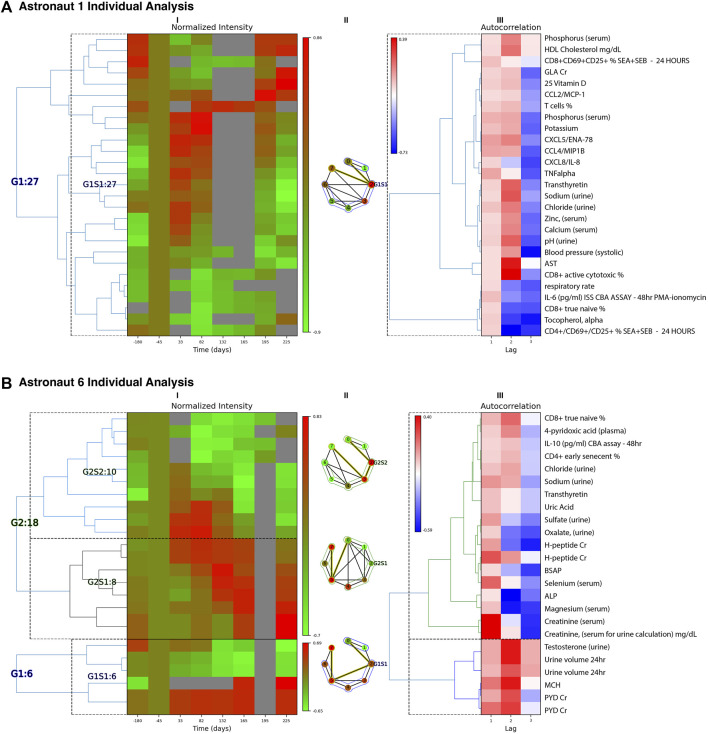
Individual Astronaut Examples. Classification results (Lag 1 patterns) are shown for astronaut 1 **(A)** and 6 **(B)**. For each astronaut, we have three panels: (I) Left heatmap: Groups/subgroups clustering of measurements, with adjusted mean time (with respect to takeoff). Times 165, 196 and 225 represent return to Earth, one and 2 months post-return respectively. (II) Middle graph: Time-resolved visibility graph representation of median intensity behavior within each group, with visibility-graph community detection identifying similar time behaviors (Zheng et al., 2021). Temporal communities are encircled and the shortest path edges are highlighted in yellow. Vertices correspond to the timepoints of the heatmap columns in (I) indexed sequentially running clockwise. (III) Right heatmap: Autocorrelation clusters and annotations for the measurements identified. The annotation labels to the right correspond to the rows of both heatmaps (I) and (III). ALP: alkaline phosphatase, BSAP: bone-specific alkaline phosphatase, CBA: cytometric bead assay, Cr: creatinine normalized, *G*: group, GLA: *gamma*-carboxyglutamic acid, MCH: mean corpuscular hemoglobin, PMA: Phorbol 12-myristate 13-acetate, PYD: pyridinium cross-links, S: subgroup, SEA: staph enterotoxin A, SEB: stap enterotoxin B*.* Multiple evaluations/repeats included.

For each time series class described above, PyIOmica’s clusterTimeSeriesCategorization was used to further resolve the time trends, by implementing two levels of agglomerative hierarchical clustering: (i) First-level group (G) clustering uses autocorrelations *ρ*_*mal*_ as a vector for computing similarities (correlation based) (ii) Second-level Subgroup (S) clustering uses the normalized time series {*Q*_*ma*_} for computing similarities. The number of groups and subgroups within each class are determined by the silhouette method (Rousseeuw, 1987). For each astronaut and classification results were then summarized in heatmaps using visualizeTimeSeriesCategorization in PyIOmica. The class [Lag 1] results from two astronauts are shown in the example outputs in Figure 2. Full plots and corresponding measurements within each class are provided in the ODFs.

### 2.5 Cross-comparison of astronaut time series

#### 2.5.1 Astronaut graph-based communities

Graph-based community analysis of the astronaut time series was performed (Zheng et al., 2022) with networkx (Hagberg et al., 2008) and scikit-network (Bonald et al., 2020). First, for each astronaut their time series periodograms *P*_*ma*_ were extracted using PyIOmica’s LombScargle method. A distance matrix with components ${\left[D_{m}\right]}_{xy}$ was computed, for each measurement *m* and astronauts *x*, *y*, where the entries correspond to the Euclidean distance between *P*_*mx*_ and *P*_*my*_. Again, a bootstrap set (50,000 signals) was constructed, and a distribution of distances between bootstrap time series was computed, with its 0.99th quantile *d*_*q*_. Then the restricted distance matrix *R*_*m*_ entries were constructed, so that distances within a radius *d*_*q*_ were set to 1, and otherwise set to zero:

$${\left[R_{m}\right]}_{xy}=\left\{\begin{array}{c} 1,\text{if }{\left[D_{m}\right]}_{xy}<d_{q}\;\\ 0,\text{if }{\left[D_{m}\right]}_{xy}\geq d_{q}\;.\; \end{array}\right.$$ (2)

Using the restricted matrices for each measurement, a weighted adjacency matrix *A* = *∑*_*m*_ *R*_*m*_ was obtained. The adjacency matrix represents a graph with astronauts as vertices and edges that connect astronauts. Hence, in the graph, an edge *E*_*xy*_ joins two astronauts *x*, *y* if there is at least one time series measurement *m* for which the pairwise distance between the periodograms *P*_*mx*_ and *P*_*my*_ was smaller than *d*_*q*_ (i.e., there is similar temporal behavior). Finally, the edge *E*_*xy*_ is given a weight that corresponds to the number of measurements for which there is temporal similarity (i.e., the number of measurements *m* with non-zero entries in the restricted distance matrix corresponding entry ${\left[R_{m}\right]}_{xy}$ , Eq. 2.

#### 2.5.2 Graph community detection

The community structure of the astronaut graph constructed above was determined: First, a graph embedding with generalized singular value decomposition (GSVD, dimension 2) of the adjacency matrix was carried out in scikit-network. The sklearn.metrics.silhouette_score of (Pedregosa et al., 2011) was used to determine the number of components based on silhouette scores (Rousseeuw, 1987). Finally, a consensus network was constructed where the procedure was repeated 1,000 times, and nodes were allocated to their highest frequency community membership (highest probability assignment).

### 2.6 Code and data availability

All data files, results and code used to generate them in this investigation, referred to as Online Data Files (ODFs) in the manuscript, and which have been reviewed per ethical approval and privacy considerations (see above), have been deposited to and are available from the NASA Life Sciences Data Archive (LSDA). LSDA has as a public-facing portal where data requests can be initiated (https://nlsp.nasa.gov/explore/lsdahome/datarequest) (see also the Data Availability Statement).

The code base for this investigation and approach are based on previous work Zheng et al. (2022), for which all material is open source and available on Zenodo (10.5281/zenodo.6751960). We note that for this investigation’s requirements the data and code are being made available through LSDA. Astronauts are under continuous scrutiny and monitoring during their missions, and additional efforts must be made to afford them the same rights as in all other (Earth-based) human subject studies. This includes all necessary precautions to ensure that personally identifiable information (PII) is not included in any public data release. Given the small number astronauts that have gone to space and participated in studies, even data for which the names are redacted/codified may still contain potentially identifiable information if not reported in aggregate. For example, knowing the length of duration, or the flight start or return to earth dates, or individual IMM report information for any astronaut may lead to their identification. Hence, all precautions must be taken to prospectively ensure that data which appear as deidentified are secured to prevent any reverse engineering that can potentially breach privacy. NASA’s IRB procedures and policies, including data restrictions, support essential steps towards ethical medical research and protection of astronaut participants (including from genomic discrimination Reed and Antonsen (2018)). The commercialization of spaceflight provides new avenues for medical human subject research, and the ethical considerations and procedures under discussion for careful future planning of research in space health (Cope et al., 2022; Griko et al., 2022).

## 3 Results and discussion

### 3.1 Astronaut cohort

We investigated time-resolved measurements from NASA astronauts across multiple missions to ISS. These astronauts had participated in two studies: the Nutritional Status Assessment and Validation of Procedures for Monitoring Crewmember Immune Function. These studies included multiple metabolic and physiological measurements (264 annotations), including blood and urine metabolite analyses and immune cellular assessments which were taken over multiple timepoints before, during, and after flight. As part of our broader investigation on astronaut integrative data analysis, we identified 47 astronauts meeting the criteria of having multiple time-resolved measurements, and of those, 38 astronauts agreed to participate in our study following informed consent for additional analyses. They had previously provided informed consent to participate in the original studies mentioned above. Out of these astronauts, 27 were found to have long-duration mission data (165 days mean), which were selected for analysis. Data were obtained for the Lifetime Surveillance for Astronaut Health (LSAH) and Life Sciences Data Archive (LSDA).

Over the course of their missions, the astronauts experienced several clinical symptoms that were provided to our team as IMM reports. We aggregated the information across astronauts and events in Table 1. Events that resolved within the first 30 days of flight are classified as space adaptation symptoms (Crucian et al., 2016). The top space adaptation events included space motion sickness (14 astronauts), headache (10), and insomnia (9). The most frequent events later in the mission, categorized as non-adaptation events, included sleep disorder (15; reported 34 times), fatigue (14), and skin rash (9).

**TABLE 1:** Integrated medical model reports.

Reported condition	Astronauts	Total events
Sleep Disorder	15	34
Space Motion Sickness (Space Adaptation)	14	14
Fatigue	14	23
Headache (Space Adaptation)	10	10
Skin Rash	9	11
Insomnia (Space Adaptation)	9	9
Skin Abrasion	8	12
Nasal Congestion	8	10
Back Sprain/Strain	8	15
Sleep Disorder due to Sleep Shift	7	7
Nasal Congestion (Space Adaptation)	7	7
Knee Sprain/Strain	7	16
Headache (CO_2_ Induced)	7	9
Allergic Reaction (Mild to Moderate)	7	11
Shoulder Sprain/Strain	6	7
Paresthesias	6	7
Space Motion Sickness Landing Prophylaxis	5	5
Eye Irritation/Abrasion	5	8
Elbow Sprain/Strain	5	10
Constipation (Space Adaptation)	5	5
Constipation	5	7
Back Pain (Space Adaptation)	5	5

As is common, not only in spaceflight but with any medical monitoring outside laboratory settings, different measurements were available for different astronauts, with highly heterogeneous and uneven time sampling, and missing data. We created a common time sampling grid across all astronauts, where measurement data for each astronaut were summarized by time averages into eight adjusted mean-based timepoints: 180 and 45 days prior to flight, early flight (33 days), mid-flight (82 days) and late flight (132 days), return to Earth (after 165 days of flight), and 30 and 60 days following return. The common time grid was used to: (i) ensure non-attributability of data back to individual astronauts, since the time of flight is sometimes unique to each astronaut, and (ii) to enable the individual measurements to be comparable across astronauts.

### 3.2 Individual astronaut time-resolved assessment

We classified each astronaut’s time-resolved measurement into different classes using spectral methods (Mias et al., 2016; Domanskyi et al., 2019; Mias et al., 2021; Zheng et al., 2022). The approach allowed the identification of sets of measurements that display time-resolved deviations from an astronaut’s baseline. This categorization provided 3 classes of time trends: (i) *Lags*, for measurements showing statistically significant autocorrelation at different lags. Here an autocorrelation of a signal at a certain lag refers to a correlation of a signal with a delayed (lagged) version of itself. (ii-iii) *Spike Maxima or Minima* for measurements that do not belong in the *Lags* class, but have punctuated intensities (spikes) that are either high (Maxima) or low (Minima) at particular timepoints. The measurements assigned to these classes are further clustered into groups (G) and subgroups (S) based on their overall autocorrelation and signal intensity similarities respectively (see Methods).

The individual astronaut signal analyses are available in the Online Data Files (ODFs). Examples of the analysis are shown in Figure 2 for two astronauts. For each astronaut there are three sets of panels shown in the figure: (I) The left panel heatmaps include the intensities of the measurements that had statistically significant (FDR < 0.05) trends for the Lag 1 class in each astronaut. The corresponding clustering of the data into groups (G) and subgroups (S) reveals similarities due to the signal autocorrelations. The corresponding autocorrelation heatmap depicted on the right of the figure as panel (III) and the labels of the measurements (rows) across both heatmaps are listed to the far right. The middle panel (II) shows for each subgroup a summary of the time-resolved median intensity structure as a graphical representation using a visibility graph (Zheng et al., 2021). Briefly, in each visibility graph the vertex sequence represents the timepoints measured (labeled by their indices starting at 0), with time moving forwards in a clockwise direction from pre-flight (index 0) to 2 months post flight (index 7). The vertex intensity indicates the median normalized intensity across measurements in the specific group/subgroup at that timepoint. The sets (communities) of vertices that show similar intensities/temporal behavior are encircled in each graph. The boundaries of these measurement communities may represent changepoints - i.e., intensity changes in sets of signals that may be linked to changes in health events. Finally, the edges of the shortest path in each graph are highlighted in yellow, and represent prominent vertices that are used in the algorithm by Zheng et al. (2021) to identify the measurement communities.

For astronaut 1, the changes associated with the Lag 1 class, shown Figure 2A, have a main trend of upregulation of a subset of immune markers early in flight (Days 33 and 82), and a return to pre-flight levels after return to Earth. The changes are associated with plasma concentrations of the cytokine CXCL5, CCL4, CXCL8, and TNF alpha, indicative of immune dysregulation (potential inflammation). Additionally, in urine there is an increase in sodium, chloride and zinc in early flight, returning to post-flight levels upon return to Earth. *γ*-carboxyglutamic acid (GLA) is decreased during flight, returning to pre-flight conditioning levels 2 months post return. GLA reflects post-translational modification of glutamic acid, and this modification is vitamin K dependent (Furie et al., 1999). On flights to the Russian space station Mir, vitamin K status in one crewmember was low during flight (Caillot-Augusseau et al., 2000) and subsequently vitamin K supplementation has been offered as a countermeasure for bone loss in astronauts. On ISS, this was not observed in a larger cohort of astronauts on longer missions (Zwart et al., 2011). In parallel, there is a decrease in immune cell levels, including CD8^+^ active and naïve relative percentages. This can be indicative of decreased immune response, as well as disruption of immune cell maturation cycles (Crucian et al., 2015).

For astronaut 6 there are 3 sets of temporal patterns, Figure 2B, with changes associated with the Lag 1 class. In the first Group, G1S1, MCH (mean corpuscular hemoglobin) and PYD (pyridinium cross-links) were found to increase during flight and stay high even following 2 months post return to earth. Increased MCH is associated with macrocytic anemia. PYD is a bone resorption marker reflecting changes in bone metabolism and bone remodeling. With a symmetrically opposite trend, urine volume and testosterone decrease in flight and remain low for up to 2 months after return to Earth. The second set, G2S1, indicates a gradual increase in intensities of H-peptide (another bone resorption marker), total and bone-specific alkaline phosphatase (BSAP), selenium, magnesium, and creatinine that persist after return to Earth. Probable indications include bone disease and kidney dysfunction, though certain values may be affected by dietary intake (e.g., magnesium). Finally, the third trend, G2S2 involves sets that show an increase in early flight but then consistent decreases in intensity that persist post flight. These include CD8^+^ T cell ‘true naïve’ and CD4^+^ T cell ‘early senescent’ relative percentages, which may indicate immune cell maturation dysregulation. IL-10 also remains reduced post flight, as compared to preflight baseline concentrations. Other compounds with similar trends include 4-pyridoxic acid, chloride and sodium in urine, transthyretin, uric acid, and sulfate and oxalate levels (in urine).

The results for all astronauts showed comparable patterns, indicative of individual-specific changes particularly associated with early spaceflight or landing stress, with multiple changes persisting up to 2 months following return to Earth. Further details and representations of all trends identified for each astronaut are available in deidentified plots and tables in the ODFs.

### 3.3 Spaceflight effects across multiple astronauts

**FIGURE 3:**
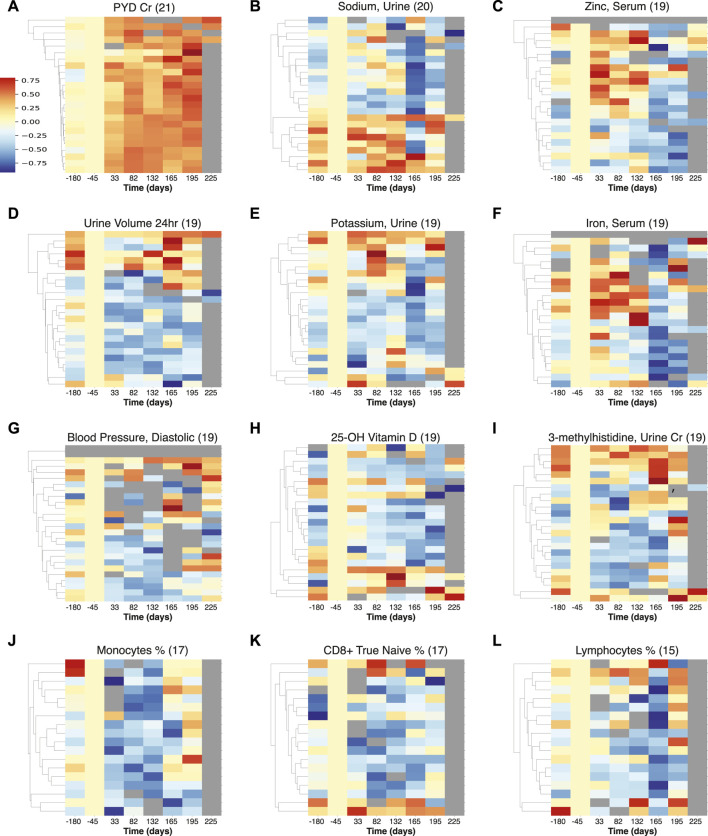
Multiple measurements visualization of temporal trends detected across astronauts. The heatmaps represent the normalized intensities of each measurement across all astronauts (rows) over time (columns). These measurements were identified to show statistically significant trends (FDR < 0.05) in multiple crew members, with the respective astronaut tallies shown in parentheses. **(A–I)** are the top measurements by frequency overall, and **(J–L)** are the top immune cell measurements. PYD: pyridinium cross links, Cr: normalized with creatinine.

To identify similarities across astronauts, we investigated how often the individual-astronaut statistically significant signals were identified across multiple astronauts. The measurements ranked by incidence (≥16), are shown in Table 2, including a trend summary and potential physiological aspects. The top examples are also visualized in Figures 3A–I across all measurements, as well as for immune cells; Figures 3J–L, and briefly discussed below.

**TABLE 2:** Aggregated measurements across astronauts.

Measurement (fluid)^***a***^	Count^***b***^	F,R, PR trends^***c*,*d***^	Physiological aspects
Pyridinium (PYD) Cr	21	*↑ ↑ ↑*	Bone resorption (Caillot-Augusseau et al., 1998; Smith et al., 1998; Smith et al., 2015)
Sodium (urine)	20	−− *↓* (*↑ ↓* −)	Saline ingestion pre-landing
Zinc (serum)	19	*↑ ↓ ↓* (*↓ ↓ ↓*)	Nausea; bone resorption (Cann and Adachi, 1983); immune response, wound healing (National Institutes of Health, Office of Dietary Supplements., 2022)
Urine volume 24 h	19	*↓ ↓ ↓* (*↓ ↑* −)	Inadequate fluid intake; fluid redistribution; renal stones (Smith et al., 2015)
Potassium (urine)	19	*↓ ↓ ↓* (*↑ ↓ ↓*)	Retention - renal stones; muscle disuse (Lane et al., 2000)
Iron (serum)	19	*↑ ↓ ↓* (−*↓ ↓*)	Iron metabolism; immune response (Dallman, 1987; Alfrey et al., 1997)
Blood pressure diastolic	19	*↓ ↑* −	Hypotension; conditioning (Fu et al., 2019)
25-OH Vitamin D	19	*↓ ↓ ↓*	Bone mass resorption (Smith et al., 2005b)
3-methylhistidine (urine) Cr	19	*↓ ↓ ↑* (−*↑* −)	Skeletal muscle maintenance (Young and Munro, 1978)
Sodium	18	*↓* − − (*↑* − *↑*)	Fluid imbalance; diet
Creatinine (serum)	18	*↓ ↓ ↓* (*↑* − −)	Muscle mass; diet (Wyss and Kaddurah-Daouk, 2000; Delanaye et al., 2017)
Chloride (urine)	18	−*↓ ↓* (*↑ ↓* −)	Dietary salt; saline ingestion (end of mission); intravenous saline at landing
Blood pressure systolic	18	*↓ ↑* −	Hypotension; conditioning (Fu et al., 2019)
Vitamin B12	17	*↓ ↓ ↓* (*↑ ↑* −)	Food absorption, neurological manifestation, ocular health (Chu and Scanlon, 2011; Green et al., 2017; Smith et al., 2021)
Transferrin receptors	17	*↓ ↓ ↑*	Iron metabolism shifts (Smith et al., 2005b)
Sulfate (urine)	17	*↓ ↓ ↓* (*↑ ↑ ↑*)	Urinary supersaturation; diet
pH (urine)	17	*↓ ↓ ↓* (*↑ ↑* −)	Kidney issues; renal stones (Smith et al., 2015)
Monocytes %	17	*↓ ↓* −	Immunity; viral reactivation
Magnesium (serum)	17	*↑ ↓* − (*↓ ↓* −)	Decreased intake; renal stones (Smith et al., 2005b)
CD8^+^ true naïve %	17	*↓ ↓ ↓*	T cell maturation dysregulation (Crucian et al., 2015)
Phosphorus (urine)	16	*↓ ↓ ↓* (*↑ ↓* −)	Renal; skeletal (Smith et al., 2005b; National Institutes of Health, Office of Dietary Supplements, 2021; Smith et al., 2021)
γ-carboxyglutamic acid Cr	16	−↑ ↑ (↓ ↓ ↑)	Vitamin K status (Furie et al., 1999; Caillot-Augusseau et al., 2000; Zwart et al., 2011)
Calcium (serum)	16	*↓ ↓ ↓* (*↓ ↑* −)	Bone metabolism; renal stone risk (Smith et al., 2015)

^*a*^Cr: normalized using creatinine.

^*b*^Count of individuals for which measurement has a statistically significant trend.

^*c*^The majority signal intensity trend is summarized with respect to preflight for: Flight (F), Return date (R), Post-return (PR) sequentially. If a secondary trend is observed it is listed in the parenthesis.

^*d*^The intensity trends are: same as preflight (−), higher intensity (*↑*), or lower intensity (*↓*).

The results from PYD are prominent and most prevalent (observed in 21 astronauts), Figure 3A, showing an increase in intensity beginning early in spaceflight, and also persisting post flight (at least for 30 days). PYD is a marker of bone resorption and is indicative of the ongoing concern of bone loss among astronauts on long-duration spaceflights (Caillot-Augusseau et al., 1998; Smith et al., 1998; Smith S. M. et al., 2005; Smith et al., 2015; Man et al., 2022). We also observe fluctuations and a decrease in urinary sodium, Figure 3B, that indicates retention - particularly upon return to Earth, and this may be related to the effects of microgravity on body fluid balance, as also seen in urine volume Figure 3D.

Zinc in serum, Figure 3C is elevated during flight, and returns to low levels after return to Earth. A pattern of increased zinc excretion in early flight and returning to lower levels later was previously reported in rats in a study of bone resorption and mineral excretion (Cann and Adachi, 1983). While zinc serum is not an indicator of nutritional zinc levels (Hennigar et al., 2018), the fluctuations may have health implications, as long term elevated zinc can induce nausea and headaches and gastrointestinal problems, potentially leading to decreased immune function and copper deficiency, while zinc deficiency could be an issue after return to Earth (National Institutes of Health, Office of Dietary Supplements., 2022).

Potassium levels were found to be generally lower (but elevated for a small subset of astronauts during flight), with a sharper decrease across astronauts upon return to earth, Figure 3E. This was also reported for short Apollo missions (Alexander et al., 1975), and the connections between muscle loss, kidney dysfunction and cardiovascular conditions need to be investigated further (Lane et al., 2000).

Iron in serum, Figure 3F, appears affected by landing events as there is a marked decrease post return to Earth indicative of potential disruption of iron metabolism, which could lead to anemia and affect the immune response (Dallman, 1987). There is also an increase in relative iron levels during flight for some astronauts, which may be related to neocytolysis (Alfrey et al., 1997). Diastolic blood pressure, Figure 3G is reduced during space flight across astronauts; this transient in-flight effect has also been reported in a smaller cohort (n = 12) (Fu et al., 2019) and could have implications for hypotension during flight (though returning to normal levels following Earth return). 25-OH Vitamin D measurements are decreased across 19 astronauts, Figure 3H, consistent with concerns relating to bone health (Smith S. M. et al., 2005). 3-methylhistidine (3-MH), normalized with creatinine (Lukaski et al., 1981), was generally decreased during spaceflight, showing some increase in levels on landing for a subset of astronauts, Figure 3I. An increase of 3-MH would be consistent with skeletal muscle degradation (Young and Munro, 1978), and the overall 3-MH changes in flight, particularly punctuated on landing require further investigation.

In terms of immune cells, we noticed a general decrease in Figures 3J–L, in monocyte, CD8^+^ naïve T cell, and “bulk” lymphocyte relative percentages. This decrease highlights the potential for immune related disorders (Kaur et al., 2005; Crucian et al., 2015; Crucian et al., 2018). This finding could also be linked to the (largely) asymptomatic reactivation of latent herpesviruses, as has previously been observed in ISS astronauts during spaceflight (Crucian et al., 2018). It should be noted that recently atopic dermatitis in some astronaut cases has been positively correlated with latent herpesvirus reactivation, in particular HSV1 (Mehta et al., 2022). Recently, Mourad et al., 2022, postulated that reactivation of VZV occurred in mothers, who experienced an asymptomatic reactivation of herpes zoster virus, similar to that described in astronauts (Mehta et al., 2004; 2014; Mourad et al., 2022).

**FIGURE 4:**
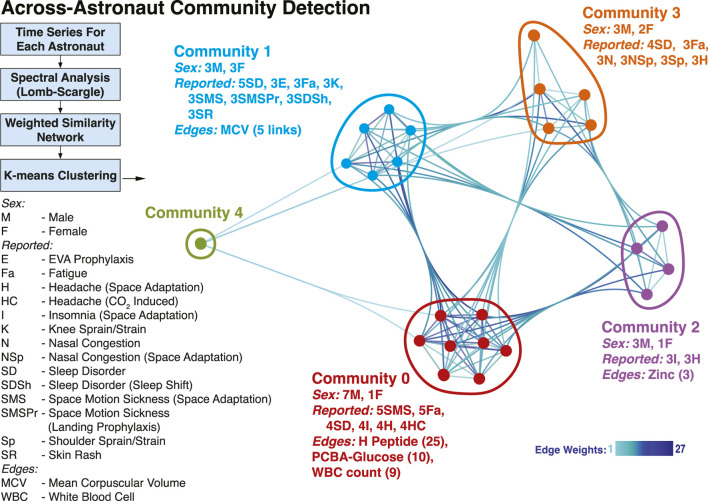
Astronaut Temporal Communities. Using the time-resolved measurements a network was constructed to identify communities of astronauts with similar temporal behavior. The nodes in the network represent astronauts, while the weighted edges correspond to different measurements, weighted with the number of astronaut pairs for which the time behavior is similar. Four Communities were identified (0–3) and a singleton, denoted in different colors. The male/female count, top reported IMM clinical symptoms, and edges (with corresponding counts/weights) associated with each community are included. Community 4 individual data are not reported for identifiability considerations.

Additionally, we investigated whether the common temporal trends across astronauts could provide a grouping of astronauts based on similar physiological responses, by studying the structure of a multi-astronaut network (Zheng et al., 2022). In the constructed network, Figure 4, nodes depict astronauts (n = 24) for which time series data were matched, while weighted edges represent how many measurements showed similarity (Euclidean distance) in their signal periodograms across pairs of astronauts. The five shown communities (labeled Community 0 through Community 4) were detected using a k-means approach, with k = 5 (see Methods). While the number of astronauts included (n = 24) restricts findings, we still observed community separations (except Community 4, which is a singleton), with distinct characteristics. The largest group, Community 0 included 7 male and 1 female astronaut. Weighted edges indicate that H Peptide drove the similarity within Community 0. During flight, the astronauts of Community 0 had reported space motion sickness predominantly (5 times) as well as sleep disorder, insomnia and headaches (4 times each). The second largest group (Community 1) is evenly balanced based on sex (3 male, 3 female). The clustering is dominated by MCV (mean corpuscular volume) behavior, and the members predominantly reported sleep disorder (5 times), as well as space motion sickness and skin rash (3 times each). Similarly, in Community 2 (3 male, 1 female) the astronauts reported insomnia and headaches (3 times each), while for Community 3 (3 male, 2 female) sleep disorder (4 times), and nasal congestion, shoulder sprain/strain and headache (3 times).

## 4 Conclusion

We have implemented an individual-focused approach to investigate personalized astronaut monitoring and detecting deviations from a wellness baseline. The astronauts experienced physiological changes that are associated with the identified molecular markers. The time-resolved analysis revealed the majority of changes to be associated with space stay, and also with the impact and stress of landing prior to readjustment to return to Earth. The impacted measurements are related to microgravity adaptation adverse effects such as bone resorption and kidney function, and to immune-system dysregulation. These changes suggest further countermeasures are necessary to prevent adverse outcomes (e.g., osteoporosis, renal stones, viral reactivation), particularly on long-duration flights. These can include enhanced food provisions, or if required, nutritional supplementation, as well as provision of relevant immune-disorder medications on board.

Our successful detection of individual-astronaut changes offers an extensible approach that can be used to integrate multiple time-resolved measurements (omics, physiological measurements, mobile device temporal data, and any temporal signal) on individual astronauts towards detecting and preventing potentially harmful medical events. By cataloguing such event onsets and associated measurement responses, the non-wellness transitions in astronauts can be used for providing timely diagnosis and countermeasures during space missions. This is vital during long-duration deep space missions, where distance from Earth necessitates self-reliance by astronaut crews to maintain their own wellness, as we expand space exploration to Mars and beyond.

## Data Availability

The data analyzed in this study are subject to the following licenses/restrictions: All data files and code used in this investigation, referred to as Online Data Files (ODFs) in the manuscript, have been deposited to and are available from the NASA Life Sciences Data Archive (LSDA). LSDA is the repository for all human and animal research data, including that associated with this study. LSDA has as a public-facing portal where data requests can be initiated (https://nlsp.nasa.gov/explore/lsdahome/datarequest). The LSDA team provides the appropriate processes, tools, and secure infrastructure for archival of experimental data and dissemination while complying with applicable rules, regulations, policies, and procedures governing the management and archival of sensitive data and information. The LSDA team enables data and information dissemination to the public or to authorized personnel either by providing public access to information or via an approved request process for information and data from the LSDA in accordance with NASA Human Research Program and Johnson Space Center (JSC) Institutional Review Board direction. Requests to access these datasets should be directed to https://nlsp.nasa.gov/explore/lsdahome/datarequest.
